# Supplementary material for: Questionnaires of interoception do not assess the same construct
Source: PLoS One. 2022 Aug 23;17(8):e0273299. doi: 10.1371/journal.pone.0273299 (PMC9397851; doi:10.1371/journal.pone.0273299)
Supplement: S1 File — (PDF) [file pone.0273299.s001.pdf]

Supporting Information 1. Factor structure of the BPQ

To explore the factor structure of BPQ, exploratory factor analysis using principal axis factoring with quartimax rotation was performed. The data set was appropriate for factor analysis (Kaiser-Meyer-Olkin test with values > 0.9 showed adequate sampling; Barlett-test of sphericity was significant). Parallel analysis indicated a single factor; also, only the first factor had an eigenvalue above 1 (14.394), This factor explained 55.4% of the total variance. All items loaded above 0.5 on this factor; factor loadings are presented in S1 Table.

S1 Table. Loadings of the individual items of the BPQ in exploratory factor analysis.

	Loadings on Factor 1 (exploratory factor analysis)
BPQ_1	0.505
BPQ_2	0.609
BPQ_3	0.678
BPQ_4	0.745
BPQ_5	0.793
BPQ_6	0.748
BPQ_7	0.739
BPQ_8	0.668
BPQ_9	0.713
BPQ_10	0.603
BPQ_11	0.689
BPQ_12	0.864
BPQ_13	0.845
BPQ_14	0.803
BPQ_15	0.846
BPQ_16	0.757
BPQ_17	0.839
BPQ_18	0.679

BPQ_19	0.645
BPQ_20	0.687
BPQ_21	0.831
BPQ_22	0.868
BPQ_23	0.793
BPQ_24	0.679
BPQ_25	0.765
BPQ_26	0.782
