# Supplementary material for: Questionnaires of interoception do not assess the same construct
Source: PLoS One. 2022 Aug 23;17(8):e0273299. doi: 10.1371/journal.pone.0273299 (PMC9397851; doi:10.1371/journal.pone.0273299)
Supplement: S2 File — (PDF) [file pone.0273299.s002.pdf]

Supporting Information 2. Exploratory factor analysis of the assessed variables

As data were appropriate for exploratory factor analysis (Bartlett's test: $p < .001$; Kaiser-Meiser-Olkin Measure = 0.839), the maximum likelihood method with oblique (oblimin) rotation was applied in order to shed more light on the overall pattern of the variables. The number of factors to be extracted was determined by parallel analysis.

Parallel analysis indicated two factors explaining 27.7% and 10.6% of the total variance, respectively. The two factors were only weakly associated (r = -0.16), factor loadings are summarized in S2 Table.

S2 Table. Loading of the variables on the two factors (values over 0.3 are marked in bold)

	Factor 1	Factor 2
Negative affect	-0.152	**0.471**
Positive affect	**0.414**	-0.139
BPQ-BA-26	**0.335**	**0.319**
BAQ	**0.504**	0.123
SSAS	0.134	**0.688**
MAIA Noticing	**0.743**	0.074
MAIA Not-Distracting	0.237	0.126
MAIA Not-worrying	0.196	**-0.528**
MAIA Attention Regulation	**0.727**	-0.194
MAIA Emotional Awareness	**0.761**	0.128
MAIA Self-Regulation	**0.635**	-0.212
MAIA Body Listening	**0.750**	0.143
MAIA Trusting	**0.532**	**-0.336**

Note. BPQ-BA-26 = Body Awareness Subscale of the short form of Body Perception Questionnaire; MAIA = Multidimensional Assessment of Interoceptive Awareness; BAQ = Body Awareness Questionnaire; SSAS = Somatosensory Amplification Scale
