# Supplementary material for: Questionnaires of interoception do not assess the same construct
Source: PLoS One. 2022 Aug 23;17(8):e0273299. doi: 10.1371/journal.pone.0273299 (PMC9397851; doi:10.1371/journal.pone.0273299)
Supplement: Attachment — Submitted filename: replies.docx [file pone.0273299.s003.docx]

Dear Reviewers,

Many thanks for your suggestions and comments on our manuscript. All issues you raised were carefully addressed in the revised version (see below). In our opinion, these changes considerably improved the overall quality of the manuscript. Hopefully, you will find the revised version appropriate for publication.

Best wishes,

Luca Vig,

corresponding author

Reviewer #1: I would like to commend the authors on a well-written manuscript. The introduction sets up contrasts between these questionnaires well. The widespread use of interoceptive sensibility as a research construct makes the understanding of convergence between measures important.

**Reply 1:** Many thanks for the positive evaluation. We also think that this is an important area that has received relatively little attention in the past.

However, I have some concerns about the analysis that was conducted in this study.

1. Information on distributions of the subscale scores are not reported. From the scatterplots it appears that there may be some skew in the measures, which is particularly apparent in the MAIA Noticing subscale. Non-normality can create artifacts in the parametric statistics that are used in this analysis. I would recommend assessing normality and using linear transformation if needed to ensure alignment between distributions and model assumptions

**Reply 2:** We decided to use Pearson correlation because of its robustness (e.g. Havlicek & Peterson, 1976; Knief & Forstmeier, 2021). In the revised version, this was changed to Spearman correlation (this change did not substantially impact the outcome of the analysis).

References:

Havlicek, L. L., & Peterson, N. L. (1976). Robustness of the Pearson Correlation against Violations of Assumptions. *Perceptual and Motor Skills*, *43*(3_suppl), 1319–1334. <https://doi.org/10.2466/pms.1976.43.3f.1319>

Knief, U., & Forstmeier, W. (2021). Violating the normality assumption may be the lesser of two evils. *Behavior Research Methods*. <https://doi.org/10.3758/s13428-021-01587-5>

____

2. The sample here is relatively small for exploratory factor analysis. I would recommend that the authors consider the role of sample size and its implications for the success of uncovering the underlying factor structure. These articles may provide useful background in this area:

Floyd, F. J. & Widaman, K. F. (1995). Factor analysis in the development and refinement of clinical assessment instruments. Psychological Assessment, 7(3), 286-299.
MacCallum, R. C., Widaman, K. F., Zhang, S., & Hong, S. (1999). Sample size in factor analysis. Psychological Methods, 4(1), 84-99.

**Reply 3:** Determination of minimum required sample size is a highly debated issue in the context of exploratory factor analysis. Certain authors recommend a minimum sample size of n = 100; also, the minimum acceptable ratio of number of participants to number of variables varies from 3 to 20 (for a review, see Mundfrom et al., 2005). As the sample size is above n = 200 and the participants to variables ratio is above 25 in our study, the data met these criteria. Still, as the bigger the better with respect to desirable sample size for EFA, this is a limitation of the study, which is mentioned in the revised version.

Reference:

Mundfrom, D. J., Shaw, D. G., & Ke, T. L. (2005). Minimum Sample Size Recommendations for Conducting Factor Analyses. *International Journal of Testing*, *5*(2), 159–168. <https://doi.org/10.1207/s15327574ijt0502_4>

____

3. The decision of how many factors to retain is crucial for this study. In the manuscript, factor retention was solely guided by the eigenvalue > 1 rule, which is known to have limitations. Typical factor analysis recommendations now include comparing fit indices of each solution, the scree test, and/or parallel analysis. Since the key outcome of this study rests on retaining the correct amount of factors, it is important that multiple metrics are used to guide factor retention. The following article provides an overview on this topic:

Fabrigar, L. R., Wegener, D. T., MacCallum, R. C., & Strahan, E. J. (1999). Evaluating the use of exploratory factor analysis in psychological research. Psychological Methods, 4, 272-299.

**Reply 4:** Many thanks for this remark. In the revised version, parallel analysis was used to determine the number of factors to be extracted (it also indicated 2 factors thus does not change the analysis).

____

4. There is no mention of whether oblique or orthogonal rotation was used (based on results, I assume oblique)

**Reply 5:** In the original version, the promax rotation with Kaiser normalization and κ = 4 in SPSS was used which is an oblique rotation. In the revised version, factor analysis was conducted with JASP, also with oblique rotation (these details are explicitly described in the revised version; see also Reply 36).

5. I could not find supporting data in the supplemental file

**Reply 6:** A supplemental file will be uploaded with the revised version.

____

6. Data collection and participant features are only briefly described. Several clarifications would be needed: Is country of residence data on participants available? Were there any cleaning procedures to screen out potentially poor quality or incomplete responses? Also, based on the questionnaire descriptions it is assumed that the survey was in Hungarian but this could also be useful in the participant description.

**Reply 7:** The Participants section of the revised version was completed with these pieces of information

____

Reviewer #2: The current study examines associations between three interoceptive sensibility measures, and positive and negative affect by means of correlations and exploratory factor analysis in a cross-sectional community sample of 265 adults. I would like to present the following questions and suggestions to the authors.

Abstract
• In the abstract, the SSAS abbreviation is used without introduction.

**Reply 8:** This is corrected in the revised version
____

Introduction
• L.44: To my opinion, the statement that interoceptive accuracy and sensibility are usually independent needs to be formulated a bit more cautiously. As it is formulated now, the statement would imply that interoceptive awareness is usually nonexistent, which is not the case. It is not because generally low correlations are found between interoceptive accuracy and sensibility (for existing measures across participants), that both can be considered independent.

**Reply 9:** Interoceptive awareness refers to the association between actual and perceived (typically measured with VAS) performance in a modality-specific sensory-behavioral task (e.g. a heartbeat perception/detection task) and indeed shows individual differences with respect to the strength of the association. However, ISb, i.e., a self-reported trait-like characteristic assessed with a questionnaire, usually shows no association with interoceptive accuracy.

____

• L.58: I am a bit confused by the use of the term “pure perception”. Perception, and interoception specifically, involves the sensing, identification, differentiation and interpretation of internal sensations, and implies by definition potential bias by associated emotions, cognitions and beliefs, for example deriving from personality factors including self-esteem and affect, as suggested by the authors.

**Reply 10:** The distinction between primary (experiential) and secondary (language-based) consciousness and processes is widely used (although admittedly not perfect). Concerning interoception, this distinction is used to differentiate between automatic (associative) and reasoning-based evaluative processes. For example, Farb and Logie (2018) make this distinction with respect to *interoceptive appraisal*, whereas Herbert and Pollatos state (2018) that *interoceptive affective evaluation* refers to the former category only. Nevertheless, the term “pure perception” was removed from the revised version and a concise description of primary and secondary consciousness is provided (“This belongs to the experiential aspect of conscious awareness, also called primary consciousness (Farthing, 1991; Köteles, 2021; Lloyd, 1989). On the other hand, various constructs also include higher levels of cognitive processing (aka secondary consciousness). For instance, attention intentionally focused on bodily sensations in order to regulate internal states or to make decisions are often part of the various ISb-related concepts (Mehling et al., 2009).”).

References:

Herbert, B. M., & Pollatos, O. (2018). The relevance of interoception for eating behavior and eating disorders. In M. Tsakiris & H. De Preester (Eds.), *The interoceptive mind. From homeostasis to awareness* (pp. 165–186). Oxford University Press.

Farb, N. A., & Logie, K. (2018). Interoceptive appraisal and mental health. In M. Tsakiris & H. De Preester (Eds.), *The interoceptive mind. From homeostasis to awareness* (pp. 227–241). Oxford University Press.

____

• L.63: It is not completely clear what the “total score” of questionnaires refers to precisely. I assume that the authors refer to the overall underlying construct assessed by questionnaires?

**Reply 11:** Thank you for this remark. The use of the term in that context was inappropriate, we removed it (“Also, ISb can be regarded as a generalization over a number of various interoceptive modalities, such as heartbeat, gastric sensations, and respiration (Khalsa et al., 2018)”).

____
• L.136 and later (continuing in the discussion): If I understand correctly, the authors argue that moderate to high correlations (e.g. between specific interoception questionnaires and positive and negative affect) imply a conceptual overlap between both. Although a correlation represents statistical overlap in terms of variance explained, it does not necessarily imply a conceptual overlap, however. At most, it may suggest a conceptual overlap.

**Reply 12:** This statement was reconsidered and reworded as follows: “These results suggest that the investigated measures of ISb might assess different (although to some extent overlapping) constructs.”

____
• L.146-151: To me, it is not clear what the precise goal is of this paragraph. Two domains are mentioned in which the BAQ is commonly used. However, the BAQ is used in much more research domains. Therefore it is not clear what the precise message here is. If the two research domains mentioned are examples, this could be made more explicit.

**Reply 13:** The paragraph is reworded in the revised version; it describes the BAQ from a more relevant point of view (“The questionnaire is widely used; it was found to be associated with well-being and positive affect in a number of studies (Daubenmier, 2005; Impett et al., 2006; Moradi & Huang, 2008; Tihanyi, Böőr, et al., 2016)”).

____
• Throughout the introduction, the authors repeatedly use the term “susceptibility”. This is not a term commonly used in the interoception literature, and to me it is not clear how it is defined by the authors. Based on what the authors write, but I may misunderstand, it seems they mean mostly “sensitivity” in this regard? Given that “susceptibility” means often more than “sensitivity” and may also imply vulnerability, the term may not be the best choice. In any case, a clear definition is required, especially in the interoception literature in which constructs are generally poorly delineated, or at least have a history of that.

**Reply 14:** The term was removed from the revised version.

____
• I would slightly disagree with the authors that the BPQ, BAQ and MAIA are currently used interchangeably by researchers. All three questionnaires are developed with their own purpose and outline the concept they are measuring in different ways. Often this concept is specified by the researchers choosing a specific questionnaire, and the choice is accounted for. However, I do agree with the statement that often all these different questionnaires are considered measuring interoception, which is fundamentally different than them being considered and used interchangeable. Researchers who aim to measure interoceptive sensibility often choose a questionnaire of the three questionnaires used here, but that does not mean they consider them being exchangeable. I do want to add that this does not take away that the message that they should not be used interchangeable (as suggested in the conclusion), is an important message.

**Reply 15:** This part was reworded as follows: “The recently used categorization of the dimensions of interoception suggests that the described dimensions are relatively homogeneous (Garfinkel, Seth, et al., 2015), thus questionnaire measures of ISb are interchangeable, i.e., they measure the same construct. In other words, ISb is not only conceptualized as a term covering different self-reported measures of interoception but (as it is assessed with questionnaires) also as a unitary construct (i.e. dimension) with various, possibly equivalent ways to operationalize. Although this is not explicitly stated and rarely investigated, papers citing Garfinkel and colleagues (2015) often build on this assumption (Eng et al., 2020; Mul et al., 2018). Based on our results, there is no reason to suppose the interchangeability of the three investigated questionnaires for the measurement of ISb.”
____
Methods
• L.225: It is not clear to me what is meant with “feedback upon request”.

**Reply 16:** The sentence was modified: “Participants could receive feedback on their scores of the eight MAIA scales and it’s explanation based on the original publication of the questionnaire (Mehling et al., 2012) following the data collection period if they requested.”

____
• L.2226: Maybe the editor could address this, but I am not sure whether the information provided on ethics approval is sufficient for PLOS ONE.

**Reply 17:** Approval number is provided in the revised version. Also, it is stated that all participants signed an informed consent form.

____
• L.232: Since the Hungarian translation of the BAQ seems to differ from the original BAQ in number of items, it may be good to add a reference (I assume Köteles, 2014) directly to this sentence to show a validated version was used.

**Reply 18:** Reference added.

____
• L. 254: I wonder what the rationale is to average scores of the MAIA g-factor scales? The average (vs. the sum) limits the range a lot.

**Reply 19:** We followed the original scoring instructions (averaging might have been used to make the subscale scores directly comparable). However, averaging (i.e. a linear transformation of the scores) is not problematic from a statistical point of view in the present correlational study.

____
• Table 1: The minimum score for the SSAS is probably a type error?

**Reply 20:** Corrected, thank you for spotting it.

____
• Is any more information available to better describe the community sample which was investigated? Also, more information on the recruitment process (e.g. how/for which purpose was the study advertised) may be helpful to better understand the studied sample.

**Reply 21:** Information about the recruitment process is provided in the revised version.

____
Results:
• It would be helpful to add the correlations in the text as well, so it becomes more clear how the strength of the correlations is interpreted. In this regard, it would also be helpful if the authors could indicate which norms or boundaries they used to assess the strength of correlations.

o For example, a correlation of 0.251 (BPQ-BA-26 and MAIA noticing) is considered weak, whereas a correlation of 0.269 (BPQ-BA-26 and SSAS) is considered weak to moderate. Or a correlation of -0.168 (BAQ – NA) is considered weak whereas a correlation of 0.154 (BPQ-BA-26 – NA) is interpreted as very weak.

**Reply 22:** In the revised version, the “classic” recommendations of Cohen (0.1: weak, 0.3: medium, 0.5: strong) were consistently used and this is stated in the statistical analysis section. Correlations were added to the text.

____
o In addition, the SSAS is mentioned to only correlate with NA and BPQ-BA-26 in the results, however also shows a correlation of 0.143 with the BAQ (which is higher than the correlation between PA and BPQ-BA-26, which is interpreted as very weak); this correlation is not discussed in the results, yet elaborated upon in the discussion.

**Reply 23:** In contrast to Pearson correlation, Spearman correlation coefficient was non-significant. In consequence, this topic was removed from the discussion.

____
o Also, it seems that the associations between BAQ and MAIA noticing/-g are not discussed in the results, as is the association between MAIA noticing and PA, yet both relationships are elaborated upon in the discussion.

**Reply 24:** These associations are mentioned in the results section of the revised version.

____
o In the results, the associations between BPQ-BA-26 and BAQ are described as weak to moderate, while in the discussion the relationship is described as particularly weak.

**Reply 25:** Particularly was omitted in the revised version (“The association between the BPQ-BA-26 and the other measures of ISb is weak”)

____
• Same goes for the interpretations of the factor loadings. Which cutoff scores were used here? For example a factor loading of 0.3 was interpreted as the BPQ-BA-26 not being part of factor 1, whereas a factor loading of 0.337 was considered indicative of the SSAS belonging to factor 2.

**Reply 26:** In the revised version, loadings above .3 were considered only.
____
Discussion:
• It is not clear to me, based on the predictions made in the introduction, why the authors describe the moderate to strong relationship between MAIA-g and BAQ (in the results described as moderate) to be remarkable.

**Reply 27:** This sentence was reworded in the revised version (“Findings indicate a moderate association between the MAIA-g and the BAQ, which suggest an overlap between the two underlying constructs (Ferentzi et al., 2020).”)

____
• The discussion of NA and its role in interoception is particularly long (l.376-412), and the link with the current findings only comes after. As a reader I felt a bit lost in this part as I did not know where the story was going.

**Reply 28:** This part of the discussion was substantially shortened.

____
• In this discussion, also the formulation of a questionnaire being “positively or negatively biased”, is unclear to me. To my opinion, based on the current research design and findings, the most accurate conclusion would be in terms of associations.

**Reply 29:** The term bias was removed from these parts of the discussion.
____
Minor suggestions:
l.223, l. 272: data were
l.229: The Body Awareness Questionnaire
l.249: The MAIA does not consist of 32 items of 8 scales. It consists of 32 items across 8 scales.

**Reply 30:** Corrected.

____

Reviewer #3: This manuscript addresses an important question. However, I have a few concerns that need to be addressed.
ABSTRACT
Typo? On should in In

**Reply 31:** Corrected.

____
Final sentence is not clear – is the conclusion that we need a new questionnaire?

**Reply 32:** This is the second (more distal) part of the conclusion, which is preceded by a more direct conclusion (“These findings suggest that the investigated questionnaires cannot be used interchangeably to assess interoceptive sensibility.”). At the end of the discussion of the manuscript, we conclude that the existing questionnaires do have their weaknesses, thus there is room for another, theoretically well-founded questionnaire.

____

INTRODUCTION
Both the question and the concepts are clearly and accurately defined in this section. My main concern is the length – it is over seven pages long. I would recommend shortening this section.

**Reply 33:** The introduction was substantially shortened.

____

Rational – some decisions seem arbitrary
(1) the choice of questionnaires needs justification. Why were these three questionnaires chosen over others? E.g., the IAS Murphy, J., Brewer, R., Plans, D., Khalsa, S. S., Catmur, C., & Bird, G. (2020). Testing the independence of self-reported interoceptive accuracy and attention. Quarterly Journal of Experimental Psychology, 73(1), 115-133, or the SCS Fenigstein A, Scheier MF, Buss AH (1975) Public and private self-consciousness: Assessment and theory. J Consult Clin Psychol 43 (4) 522–527 , or the BMQ Burg, J. M., Probst, T., Heidenreich, T., & Michalak, J. (2017). Development and psychometric evaluation of the body mindfulness questionnaire. Mindfulness, 8(3), 807-818. I’m sure there are others……………..

**Reply 34:** The choice was justified in the revised version (and the limited number of included questionnaires is mentioned among the limitations of the study): “Keeping in mind the aforementioned aspects, now we will take a closer look at three widely used constructs that fit the definition of ISb and the questionnaires designed to assess them: the BPQ-BA, the Body Awareness Questionnaire (BAQ) (Shields et al., 1989), and the MAIA. Besides their popularity, we chose these questionnaires because each of them represents a distinct approach to grasp ISb. The BPQ-BA has a unique approach as long as it focuses on relatively simple body sensations, without involving interpretation. The BAQ is an example of questionnaires that aim to assess normal and non-emotive bodily processes. Last but not least, none of the other measures aims to grasp as many aspects of interoception, as the MAIA.”

____

(2) Additionally, what was the reason for focusing on the noticing and ‘g’ scales of the MAIA? You mention in the introduction that the emotional awareness scale had previously been associated with the other questionnaire ……… yet you chose not to focus on the individual scales? This needs more justification. The ‘g’ scale seems to confound a range of different interoceptive constructs. I would recommend considering the scale separately – afterall, the aim of the present paper is to highlight to researchers that such differences need to be considered?

**Reply 34:** We think that the g-scale does not confound a range of different constructs but represents an overarching factor behind those constructs. In other words, the differentiation of the six subscales is not really justified, as shown by Ferentzi et al., 2020 (“However, a recent paper showed that the most prominent component of the construct can be well grasped with an overreaching factor (called MAIA-g in this paper) that includes six out of eight factors, while the remaining two (Not-Worrying and Not-Distracting) are independent (Ferentzi et al., 2020).”). Thus, the use of the g-scale appears more appropriate than the inclusion of the separate scales. The only exception is the Noticing scale, because it measures primary experience (“Concerning the level of cognitive processing, only the Noticing subscale refers to the direct body experience, and, according to Mehling (2016), it is the most similar to the earlier questionnaires (and concepts) of bodily awareness.”).

____

(3) My understanding of The Somatosensory Amplification Scale is that previous research has found it lacks internal reliability. One potential reason is that it may measure more than one construct. As teasing apart body related constructs was the aim of the present paper why use this scale to compare the others against?

**Reply 35:** We had two reasons. First, the SSAS was used in the validation process of the BPQ-BA and their positive association was interpreted as indicator of convergent validity. Second, somatosensory amplification is a construct that is very close to ISb (actually, it fits the definition of Garfinkel et al (2015, p.67): “the self-perceived dispositional tendency to be internally self-focused and interoceptively cognisant”; this is explicitly stated in the revised version), but usually does not considered an aspect of it, mainly because of its association with NA. This makes it an excellent anchor point for our study, which attempts to explore the associations between indicators of ISb and PA/NA.

____
ANALYSIS
It might be problematic to add the noticing scale of the MAIA and ‘g’ together into the factor analysis when ‘g’ already contains the noticing scale………again this might be a reason to include each of the MAIA scales separately. Additionally, should the individual items, rather than the scales, go into the factor analysis?

**Reply 36:** The size of the sample does not allow the inclusion of individual items into the FA. To address the issue with the noticing subscale and the g-scale, we rerun the factor-analysis with a g-scale that does not contain the four items of the Noticing subscale (the correlation between the full and this shortened g-scale is .99).

____
DISCUSSION
The discussion is again very long and reads like a review of questionnaire measures rather than a discussion of the findings. While it raises a number of interesting points the focus should be on the present results.

**Reply 37:** Similarly to the introduction, the discussion was substantially shortened in the revised version (see also Reply 28).

____
I am not sure I agree with the statements about an ideal interoception questionnaire L 459-464. Surely there is no such thing as an ‘ideal’ questionnaire, rather researchers should carefully select their questionnaire based on their research question and the underlying interoceptive construct they want to measure. I am not sure it makes conceptual sense to entirely separate interoception and affect as suggested in point 2 in this section. I am also not sure that an ‘ideal’ questionnaire would exclude the evaluative component – surely this is an important component, and again, whether it is assessed will depend on the research question.

**Reply 38:** We think that the entire framework of Garfinkel and colleagues (2015) relies on the sensory aspect of interoception. We formulated our recommendations with keeping this approach in mind. This does not mean that the evaluative aspect is not important or can be completely separated from the sensory. This is elaborated in the discussion of the revised version.
