# Supplementary material for: Questionnaires of interoception do not assess the same construct
Source: PLoS One. 2022 Aug 23;17(8):e0273299. doi: 10.1371/journal.pone.0273299 (PMC9397851; doi:10.1371/journal.pone.0273299)
Supplement: Attachment — Submitted filename: Response_to_reviewers.docx [file pone.0273299.s004.docx]

Dear Dr. Grynberg,

Thank you for the suggestions and comments on our manuscript. All issues you raised were carefully addressed in the revised version (see below). In our opinion, these changes considerably improved the overall quality of the manuscript. Hopefully, you will find the revised version appropriate for publication.

Best wishes,

Luca Vig,

corresponding author

_________________________

Editor’s comments

1. In the introduction and the method sections, could you provide more information/illustrations about the BAQ (i.e., items)

**Reply 1**: Additional items were included in the introduction; also, description of the BAQ in the methods section was somewhat extended.

2. Please discuss the low internal reliability of the SSAS:

**Reply 2**: This issue is discussed among the limitations of the revised version (“Fourth, the internal consistency of the SSAS was quite low. Cronbach’s alpha values in this domain are commonly reported in the literature and might reflect the conceptual heterogeneity of the construct (for a detailed discussion, see Köteles & Witthöft, 2017).”).

3. I agree with R3 about making your main objective clearer: evaluating the associations between the 3 questionnaires to examine their particularities (rather than proposing guidelines to develop a new questionnaire).

**Reply 3**: Guidelines for a novel questionnaire were removed from the revised version.

4. I agree with R3 about the different factors the MAIA because you clearly mentioned in the introduction its multi-dimensionality. Removing the two more “maladaptive scales” is a bit surprising and considering each subscale may provide relevant information. I would conduct correlations with all MAIA subscales

**Reply 4**: In the revised version, all MAIA scales are included.

5. I would suggest to remove the exploratory analysis if the sample size is too small to make appropriate analyses or to increase the sample size.

**Reply 5**: As reviewer 3 explicitly asked us to include a modified version of the factor analysis (see Reply 11), we present it as supplementary material. The analysis includes all MAIA subscales but does not include the MAIA_g. As the outcome of this analysis does not differ from that of our original analysis (using the MAIA Noticing and MAIA_g only), we completely removed the original analysis.

6. The discussion could indeed more specifically focus on the latent constructs of ISb questionnaires/dimensions based on the correlations (the phrasing issues could be addressed in the limit section). You could also for instance add recommendations about which questionnaires/subscales should be considered in future studies depending on the research objective

**Reply 6**: Recommendations for future studies are included (“As the questionnaires included in this study do not measure the same construct, authors of future studies should carefully consider the distinctive features of the individual measures in order to choose the scale that best suits their needs. To assess the perceived ability to sense signals originating from within the body (i.e. primary percepts), the MAIA Noticing appears to be the best option. The BPQ-BA also measures direct experience, but its emphasis on sympathetic activation-related sensations makes it more appropriate for the investigation of the subjective aspects of stress. The BAQ might be the primary choice to explore the background of the association between body awareness and positive affect, including the direction of causality. Finally, as the majority of the MAIA subscales includes affective and/or cognitive evaluation (e.g. meaning) of the perceived sensations, they appear usable in studies that focus on the evaluative aspect of interoception.”).

Concerning the paragraph on the phrasing issues, we think that this is not a limitation of the current study, but a more general issue impacting all kinds of primary percepts. As it is rarely discussed or even mentioned in the recent literature, we feel that is worthy of mentioning.

7. I understand the relevance to include negative affect but please better justify/discuss the implications of the associations between the ISb questionnaires and PA.

**Reply 7**: The impact and importance of PA is discussed in more detail in the revised version (“With respect to an adaptive interpretation, noticing and appreciating the messages of the body can improve positive affect and subjective well-being. On the other hand, better psychological functioning enables people to allocate more attentional resources to various stimuli, including information originating in the body (Ferentzi et al., 2019).”; “The BAQ might be the primary choice to explore the background of the association between body awareness and positive affect, including the direction of causality.”).

8. Conclusion: a word is missing ‘cannot be..to Isb’

**Reply 8**: Thank you for spotting this error, it was corrected (“Thus, one has to keep in mind that results obtained with a certain questionnaire of interoception cannot be generalized to ISb.”)

______

Reviewers' comments:

Reviewer #1: The authors have addressed all of my comments regarding the manuscript. I recommend the paper for publication; however, I strongly suggest that the authors closely check the writing for grammar before going to press. There is also formatting inconsistency in use of decimal points or commas to separate the integer and fractional part of numbers.

**Reply 9**: Many thanks for the positive evaluation. The mentioned issues were addressed in the revised version.

Reviewer #3:
Final sentence is not clear – is the conclusion that we need a new questionnaire?
Reply 32: This is the second (more distal) part of the conclusion, which is preceded by a more direct conclusion (“These findings suggest that the investigated questionnaires cannot be used interchangeably to assess interoceptive sensibility.”). At the end of the discussion of the manuscript, we conclude that the existing questionnaires do have their weaknesses, thus there is room for another, theoretically well-founded questionnaire. ____
I didn’t have any concerns about the conclusion that the questionnaires should not be used interchangeably. You have provided data that support that conclusion. What is not clear is why you review the limitations of each questionnaire and conclude that a new questionnaire is needed. This is going beyond the aim of your study which was to see if the questionnaire measures the same underlying latent construct. If you want to critically review the interoception questionnaires, then this is a different aim and should be a different paper. I maintain that the final sentence of the abstract does not tally up to the aim of the study.

**Reply 10**: The final sentence recommending the development of a new questionnaire was removed from the conclusion.

Additionally, what was the reason for focusing on the noticing and ‘g’ scales of the MAIA? You mention in the introduction that the emotional awareness scale had previously been associated with the other questionnaire ……… yet you chose not to focus on the individual scales? This needs more justification. The ‘g’ scale seems to confound a range of different interoceptive constructs. I would recommend considering the scale separately – afterall, the aim of the present paper is to highlight to researchers that such differences need to be considered?
Reply 34: We think that the g-scale does not confound a range of different constructs but represents an overarching factor behind those constructs. In other words, the differentiation of the six subscales is not really justified, as shown by Ferentzi et al., 2020 (“However, a recent paper showed that the most prominent component of the construct can be well grasped with an overreaching factor (called MAIA-g in this paper) that includes six out of eight factors, while the remaining two (Not-Worrying and NotDistracting) are independent (Ferentzi et al., 2020).”). Thus, the use of the g-scale appears more appropriate than the inclusion of the separate scales. The only exception is the Noticing scale, because it measures primary experience (“Concerning the level of cognitive processing, only the Noticing subscale refers to the direct body experience, and, according to Mehling (2016), it is the most similar to the earlier questionnaires (and concepts) of bodily awareness.”). ____

I still disagree with this. There are now plenty of studies that find some sub-scales of the MAIA relate to particular disorders, while others don’t. This implies they are not measuring the same thing. I would like to see a factor analysis in supplementary information using the individual scales.

**Reply 11**: This analysis is presented as supplementary material in the revised version.

(3) My understanding of The Somatosensory Amplification Scale is that previous research has found it lacks internal reliability. One potential reason is that it may measure more than one construct. As teasing apart body related constructs was the aim of the present paper why use this scale to compare the others against?
Reply 35: We had two reasons. First, the SSAS was used in the validation process of the BPQ-BA and their positive association was interpreted as indicator of convergent validity. Second, somatosensory amplification is a construct that is very close to ISb (actually, it fits the definition of Garfinkel et al (2015, p.67): “the self-perceived dispositional tendency to be internally self-focused and interoceptively cognisant”; this is explicitly stated in the revised version), but usually does not considered an aspect of it, mainly because of its association with NA. This makes it an excellent anchor point for our study, which attempts to explore the associations between indicators of ISb and PA/NA. ____

This response doesn’t really address my question about reliability?

**Reply 12**: This issue was addressed in the limitations section as follows:“Fourth, the internal consistency of the SSAS was quite low. Cronbach’s alpha values in this domain are commonly reported in the literature and might reflect the conceptual heterogeneity of the construct (for a detailed discussion, see Köteles & Witthöft, 2017).”

ANALYSIS It might be problematic to add the noticing scale of the MAIA and ‘g’ together into the factor analysis when ‘g’ already contains the noticing scale………again this might be a reason to include each of the MAIA scales separately. Additionally, should the individual items, rather than the scales, go into the factor analysis?
Reply 36: The size of the sample does not allow the inclusion of individual items into the FA. To address the issue with the noticing subscale and the g-scale, we rerun the factor-analysis with a g-scale that does not contain the four items of the Noticing subscale (the correlation between the full and this shortened g-scale is .99). ____

Irrespective of the correlation it is still inappropriate to the noticing scale and the g scale (including noticing) into the same factor analysis as this would inflate the chances of the two loading onto the same factor. If removing noticing from the g scale does not influence the results then why not report that analysis? It would be more appropriate?

**Reply 13**: The factor analysis has been changed (see Reply 11). It does not contain the g-scale and does include all MAIA scales.

DISCUSSION The discussion is again very long and reads like a review of questionnaire measures rather than a discussion of the findings. While it raises a number of interesting points the focus should be on the present results. Reply 37: Similarly to the introduction, the discussion was substantially shortened in the revised version (see also Reply 28). ____ I am not sure I agree with the statements about an ideal interoception questionnaire L 459-464. Surely there is no such thing as an ‘ideal’ questionnaire, rather researchers should carefully select their questionnaire based on their research question and the underlying interoceptive construct they want to measure. I am not sure it makes conceptual sense to entirely separate interoception and affect as suggested in point 2 in this section. I am also not sure that an ‘ideal’ questionnaire would exclude the evaluative component – surely this is an important component, and again, whether it is assessed will depend on the research question. Reply 38: We think that the entire framework of Garfinkel and colleagues (2015) relies on the sensory aspect of interoception. We formulated our recommendations with keeping this approach in mind. This does not mean that the evaluative aspect is not important or can be completely separated from the sensory. This is elaborated in the discussion of the revised version.

The discussion still seems to go well beyond a discussion of the present results and the aim. Indeed, there are sections where your results are not referred to. In terms of Garfinkels definition, two scales recently developed by Murphy et al. seem to have taken care of that question. The Interoceptive Accuracy Scale and the Interoceptive Attention Scale. Please comment on what you think is needed beyond these two well validated and conceptualised scales.

**Reply 14**: Parts referring to the development of an ideal questionnaire were removed.
