# Supplementary material for: Questionnaires of interoception do not assess the same construct
Source: PLoS One. 2022 Aug 23;17(8):e0273299. doi: 10.1371/journal.pone.0273299 (PMC9397851; doi:10.1371/journal.pone.0273299)
Supplement: Attachment — Submitted filename: Response to Reviewers.docx [file pone.0273299.s005.docx]

Dear Dr. Aspell, dear Reviewers,

Thank you for the suggestions and comments on our manuscript. All issues you raised were carefully addressed in the revised version (see below). We believe, these changes considerably improved the overall quality of the manuscript. Hopefully, you will find the revised version appropriate for publication.

Best wishes,

Luca Vig,

corresponding author

Reviewer #4: Thank you for the opportunity to review this research, particularly at this late stage in the peer-review process. Having read through the paper, and the dialogue between the authors and the other reviewers, I have some suggestions to add which may assist the authors in publishing the work, and making a useful contribution to the literature.

It is unfortunate timing perhaps, but I found the statement in the Introduction (and the premise of the paper) “Although the implicit assumption is that these questionnaires assess basically the same (or at least highly similar aspects of the same) phenomenon, this has not been systematically investigated to date” – to be incorrect. In fact, Desmedt, Heeren, and colleagues (2022) recently considered the associations between five self-report measures, including the three measures focused on in the present work (the BAQ, the BPQ and the MAIA). Given that Desmedt, Heeren, and colleagues (2022) used a much larger sample (n = 1003), a wider pool of measures, and more sophisticated set of analyses, I am not sure what the present work adds with regards to the associations between measures of interoception (particularly as the paper used a novel Hungarian translation of the BPQ, which has not been fully validated). The correlational analyses alone are not a strong assessment of the underlying associations between the measures.

To that end, my suggestion is that the authors refocus the paper in one of two ways:
• Focus on the reporting the validation of the Hungarian translation of the BPQ (see Swami & Barron, 2019 for reporting and analytic guidelines). I recommend that the authors collect more data if they pursue this option.
• Focus on the relationships between the interoception measures and the indices of positive and negative affect. For example, it would be interesting to see hierarchical regression analyses that identify the variance in positive/negative affect that is uniquely accounted for by each questionnaire measure. This could support the authors’ assertion that the questionnaire measures are incrementally distinct.

**Reply 1** We don't think that timing (which often depends on the availability of the reviewers, as in the case of this paper) should be a concern here. Our study has been planned well before the mentioned article was published. The fact that two different studies using different samples and analyses has similar results underlines the validity of them both. We believe even if one single empirical study (not without weak points) found something, it is still worth investigating the same question using another sample and method. In the era of open science, we think it should be ok to have similar papers published a few months apart.

As for the practical part, we focus a bit more on the associations between the assessed indicators of ISb and positive and negative affect (including regression).

General
I suggest that the authors have the manuscript proofread for grammatical issues, which will enhance the clarity of the work.

**Reply 2** The manuscript has been carefully proofread.

Abstract
Line 1 – the phrasing of this sentence is not clear. I know you mean that questionnaire measures are sometimes referred to as interoceptive sensibility, but it looks as though you are saying that interoception itself is sometimes called interoceptive sensibility.

**Reply 3** Thank you for pointing out this issue, it was corrected. („There are a number of questionnaires assessing the self-reported trait-like aspect of interoception, also called interoceptive sensibility (ISb).”)

Introduction
The authors focus heavily on Garfinkel and colleagues’ (2015) tripartite model of interoception. However, more recent models (e.g., Desmedt, Luminet et al., 2022; Khalsa et al., 2018) might be a more appropriate and useful theoretical underpinning for distinguishing between the different components of interoception the authors purport to be measured by the BAQ, BPQ and MAIA.

**Reply 4** The intorduction was extended with more recent models. (see the definition (first paragraph of the Introduction) and the refinement of the taxonomy following the model of Garfinkel and colleagues (2015) (second paragraph of the Introduction))

Methods
Briefly comment on the I-PANAS-SF Hungarian valiation. i.e., did it support the original factor structure?
I recommend computing McDonald’s omega instead of Cronbach’s alpha for these questionnaire measures, as it performs more reliably.

**Reply 5** Factor structure of the used version of the PANAS was confirmed by CFA (Gyollai et al., 2011). In the revised version, McDonald’s omega is used as indicator of internal consistency.

Limitations
The present findings may be constrained by linguistic or local contextual factors, which might influence the understanding/meaning of the latent constructs, and potentially limit generalisability (for discussions, see Ma-Kellams, 2014; Todd, 2020; see also Swami & Barron, 2019).

**Reply 6** We added this point to the limitations. **(„**Fifth, cultural and linguistic factors might also limit the generalizability of the findings (for discussion see Ma-Kellams, 2014).”)

Table 1
Why were some scores computed for some measures, and means for others? Consistency in computing means would facilitate comparison for readers across the measures.

**Reply 7** We always followed the formula used by the authors of the original questionnaire. This is always useful if one want to compare findings with those of other studies. As the present study is interested in associations between constructs, differences in the calculation of total scores (i. e. sum of item scores or average) do not impact the results.
