# Supplementary material for: Questionnaires of interoception do not assess the same construct
Source: PLoS One. 2022 Aug 23;17(8):e0273299. doi: 10.1371/journal.pone.0273299 (PMC9397851; doi:10.1371/journal.pone.0273299)
Supplement: Attachment — Submitted filename: Response to Reviewers.docx [file pone.0273299.s006.docx]

Dear Dr. Aspell, dear Reviewers,

Thank you for all your comments and suggestions. We addressed the issues raised; hopefully, you will find the revised version appropriate for publication.

Best wishes,

Luca Vig,

corresponding author

Reviewer #4: Thank you for the opportunity to review this work again. The authors have addressed most of my comments thoroughly. I have just a few remaining minor suggestions, following which I believe the work will be suitable for publication.

I refer to the editor for guidance on PLos referencing style, which appears incorrect.

**Reply 1** Thank you for noticing this mistake, we have changed the reference style from APA 7^th^ edition to Vancouver style using Zotero. We checked and made sure that the reference list is complete and correct as requested. To our knowledge, none of the cited articles have been retracted.

Participants:
- Did participants receive any payment?

**Reply 2** We added this information („Participants did not receive any payment; but they could receive feedback on their scores of the eight MAIA subscales and their explanation based on the original publication of the questionnaire (Mehling et al., 2012) following the data collection period if they requested.”

- What was deemed to be a ‘high proportion of missing items’? e.g., >20%?

**Reply 3** We specified this issue. (“Originally, 392 individuals started to fill out the questionnaire; 127 of them were excluded because they quit before completing the entire test battery.”)

- Is data available for additional participant characteristics that would better characterise the representativeness of the sample, E.g., occupation status etc.?

**Reply 4** We added information about the level of education. (“In terms of educational qualification, 78,5% reported higher education (university degree), 20,8 % secondary level education (high school), and less than 1 % primary level education.”)

Line 392 tacked changes document, ‘details’ should be changed back to ‘detail’

**Reply 5** Thank you for pointing out this mistake, we corrected it.

Supplementary materials
In line with my previous review comments, I have some concerns about the novel psychometric validation of the BPQ (I recommend further data collection for a full validation, if possible).
- The authors do not indicate if or how they divided the sample for the EFA and CFA analyses (it is not appropriate to conduct EFAs and CFAs using the same sample). See Worthington & Whittaker (2006) or Swami and Barron (2019) for sample size recommendations for EFA based on item communalities. Given the small sample, I recommend prioritising EFA over CFA (see Swami et al., 2021), and recommending that CFA is performed in the future in the Discussion.
- Principal axis would be preferable to maximum likelihood (see Watkins, 2018; Statistical simulations have found that PA outperforms ML when the relationships between measured variables and factors are relatively weak (≤.40), sample size is relatively small (≤300), multivariate normality is violated, or when the number of factors underlying the measured variables is misspecified)
- What rotation was used for the EFA? (e.g., Quartimax, given the expectation of a single orthogonal factor?)
- How did you determine the number of factors to extract in the EFA? (e.g., eigenvalues >1, parallel analysis, examination of scree plot, minimum average partials, etc.?) I recommend parallel analysis or MAP

**Reply 6**: CFA was removed, EFA was performed using your recommendations. The text was corrected as follows: “To explore the factor structure of BPQ, exploratory factor analysis using principal axis factoring with quartimax rotation was performed. The data set was appropriate for factor analysis (Kaiser-Meyer-Olkin test with values > 0.9 showed adequate sampling; Barlett-test of sphericity was significant). Parallel analysis indicated a single factor; also, only the first factor had an eigenvalue above 1 (14.394), This factor explained 55.4% of the total variance. All items loaded above 0.5 on this factor; factor loadings are presented in S1 Table.”
